# Multispecific Organic Cation Transporter 1 (OCT1) from *Bos taurus* Has High Affinity and Slow Binding Kinetics towards Prostaglandin E2

**DOI:** 10.1371/journal.pone.0152969

**Published:** 2016-04-05

**Authors:** Xiao He, Denisse Garza, Sanjay K. Nigam, Geoffrey Chang

**Affiliations:** 1 Skaggs School of Pharmacy and Pharmaceutical Sciences, University of California San Diego, La Jolla, CA 92093, United States of America; 2 Department of Pediatrics, University of California San Diego, La Jolla, CA 92093, United States of America; 3 Department of Medicine and Cellular and Molecular Medicine, University of California San Diego, La Jolla, CA 92093, United States of America; 4 Department of Pharmacology, University of California San Diego, La Jolla, CA 92093, United States of America; University of Cambridge, UNITED KINGDOM

## Abstract

Organic cation transporter 1 (OCT1, SLC22A1), like many solute carrier 22 (SLC22) family members, is important for the disposition of clinically important drugs, metabolites and signaling molecules. Several studies suggest that SLC22 family (eg. organic anion transporters or OATs and OCTs) bind and possibly transport prostaglandins with relatively high affinity (submicromolar). The affinities of OCT1 and OATs toward PGE2 and PGF2a reported in these cell-based transport studies are considerably greater than for xenobiotics and natural metabolite substrates—in many cases over 100-fold higher. This raises the possibility that prostaglandins are key endogenous substrates and/or that they act on the transporter in a manner different from other substrates such as xenobiotics and lower affinity metabolites. To further investigate OCT1—prostaglandin interactions, we designed biophysical studies using purified bovine OCT1 (*Bos taurus*, btOCT1/SLC22A1) with PGE2 analogs, in fluorescently labeled and label-free formats. Using fluorescence polarization (FP), we detected a binding of btOCT1 to the PGE2-Rhodamine conjugate at submicromolar affinity, consistent with affinity data for PGE2 from cells over-expressing the related human OCT1. Using purified native btOCT1 as analyte and biotinylated PGE2 analog as ligand, our data from surface plasmon resonance (SPR) revealed that btOCT1 specifically interacts to PGE2 with K_D_ values in the hundred nanomolar range. BtOCT1 also demonstrated a slow association (k_a_) in the range of 10^3^ M^-1^s^-1^ and an even slower dissociation rate (k_d_) in the range of 10^−4^ s^-1^ for PGE2, suggesting the possibility of a different mode of binding compared to other structurally unrelated transported substrates of low-affinity (eg. drugs, metabolites). Our results complement *in vitro* transport studies and provide direct evidence that OCT1—which is normally expressed in liver and other tissues—interacts with prostaglandin analogs. While it is not entirely clear from the published literature whether OCTs function as major prostaglandin transporters, the tight binding of the naturally occurring PGE2, as well as the slow dissociation rate, could conceivably affect the transport of lower affinity substrates such as drugs and metabolites by SLC22 transporters. More research is necessary to establish the extent to which individual SLC22 family members actually function as PG transporters *in vitro* and *in vivo* and to investigate whether PGs can, independent of being directly transported, alter the ability of SLC22 transporters to handle drugs and other substrates.

## Introduction

A number of SLC and ABC transporters have broad substrate specificities and are able to transport a wide range of drugs and endogenous metabolites, as well as exogenous and endogenous toxins [1–6]. Because they are of substantial pharmacological importance–particularly for the absorption, distribution and elimination of drugs–they are often referred collectively as “drug transporters” [1, 2]. Several of these transporters include members of the SLC22 family, which has over two dozen members, among which are organic cation transporters (OCTs) and anion transporters (OATs). OCTs/OATs have received significant attention by pharmaceutical companies and regulatory agencies because of their roles in drug disposition and drug-drug interactions [1, 6–11].

A considerable amount of evidence also suggests a role for these transporters in normal metabolism and in metabolic diseases [1] (Table 1). This includes investigations of the binding and transport of key metabolites and signaling molecules, such as cyclic nucleotides, polyamines and prostaglandins. Historically, there has been little research on the role of these “drug” transporters in regulating or modulating cellular signaling pathways and their potential mechanistic involvement in mammalian cell behavior. However, this notion is changing [1, 8, 9]. For example, MRPs (ABCC family) appear to play a role in regulating cellular cAMP levels and affect cellular function [12, 13]. Furthermore, it has been argued that the general focus on drug substrates for these transporters has been somewhat misleading from a physiological standpoint, and SLC and ABC multispecific “drug” transporters are likely critical for small molecule remote communication (“remote sensing and signaling”) between cells, tissues, organs and organisms [1, 6, 8, 9].

**Table 1:** OCT1 and natural metabolites interactions characterized by various studies.

Ligand Name	Type	KD (μM)	ka (M^-1^s^-1)^	kd (ms^-1^)	Km (μM)	Ki	IC50 (μM)	Reference
Agmatine	Metabolite				8.6	23926	24000 (0.1 MPP)	Grundemann 2003
Choline	Metabolite					3450	3450 (0.05 TEA)	Bednarczyk 2003
Dopamine	Drug; Metabolite					487	487 (0.05 TEA)	Bednarczyk 2003
Histamine	Drug; Metabolite					3007	3007 (0.05 TEA)	Bednarczyk 2003
N1-Methyl-nicotinamide	Metabolite					1035	1035 (0.05 TEA)	Bednarczyk 2003
Thiamine	Drug; Metabolite					434	434 (0.05 TEA)	Bednarczyk 2003
Tyramine	Metabolite					107	107 (0.05 TEA)	Bednarczyk 2003
Acetycholine	Metabolite					576	580 (0.2 MPP)	Lips 2005
Nicotine	Drug; Metabolite					185	186 (1 TEA)	Lips 2005
Prostaglandin E2	Metabolite (Hormone)				0.66			Harlfinger 2005
		0.54						He (This Study, FP)
		0.10	4837*	0.47*				He (This Study, SPR*)
Prostaglandin F2a	Metabolite				0.48			Harlfinger 2005
B-Estradiol	Drug; Metabolite (Hormone)					6	5.7 (0.025 MPP)	Hayer-Zillgen 2002
Progesterone	Drug; Metabolite (Hormone)					3	3.1 (0.025 MPP)	Hayer-Zillgen 2002
Serotonin	Metabolite					19984	>20000 (0.025 MPP)	Amphoux 2006
Cyclo(His-Pro)	Metabolite				655			Taubert 2007
Tetramethylammonium	Metabolite					2071	2071 (1 Metformine)	Choi 2007
Butylguanidine	Metabolite					205	210 (5 TEA)	Kimura 2009
Dimethylguanidine	Metabolite					528	540 (5 TEA)	Kimura 2009
Guanidinosuccinate	Metabolite					1506	1540 (5 TEA)	Kimura 2009
Guanidinovalerate	Metabolite					646	660 (5 TEA)	Kimura 2009
Methylguanidine	Metabolite					2310	2360 (5 TEA)	Kimura 2009
Phenylguanidine	Metabolite					225	230 (5 TEA)	Kimura 2009
Propylguanidine	Metabolite					352	360 (5 TEA)	Kimura 2009
Tetramethylguanidine	Metabolite					470	480 (5 TEA)	Kimura 2009

* Average between two parallel kinetics analyses.

Prostaglandins have well described roles in physiology [14–16]. In the kidney, the transport of prostaglandins, thought to be mediated in substantial part by SLC transporters, including members of SLC22, is important for normal proximal tubule cell function, where it has effects on blood flow as well as the handling of sodium and water by kidney [14]. The complex systemic response to endotoxins, including pyrexia and other changes, is regulated by prostaglandins [15]. Furthermore, the inactivation of PGE2 signal is thought to occur primarily in liver, lung, kidney and other tissues by carrier-mediated cellular uptake and subsequently enzymatic oxidation [16]. SLC22 transporters likely have a modulatory role on prostaglandin levels at the tissue and/or systemic levels.

However, a direct interaction between PG and purified SLC22 transporter has yet to be established. In general, assays used to demonstrate ligand interaction with a transporter have been done *in vitro* involving either mammalian cell lines or frog oocytes over-expressing the transporter (eg. OCT1, OAT1 and other SLC transpoters) *ex vivo* assays (eg. tissue slices or organ culture) and *in vivo* knockout data in mice lacking a drug transporter (eg. one of the SLC22 family members) frequently help complement the *in vitro* data for certain ligands [17–20]. Interestingly, knockout studies increasingly support a major physiological role for SLC22 “drug” transporters such as OCTs and OATs [1, 18, 19, 21–23].

Nevertheless, these different methods, while interpreted from the perspective of ligand transport via the transporter, do not specifically address direct binding of the ligand to purified transporter protein. In this study, we expressed and purified a stable, tag-free bovine OCT1 to characterize its interaction with PGE2 directly. A fluorescently labeled PGE2 (PGE2-Rhodamine) enabled us to observe a direct binding between btOCT1 and PGE2. We also establish, for the first time, a method using SPR to characterize the binding kinetics of purified label-free btOCT1 to PGE2. While considerable data indicates that certain SLC22 family members (eg. OATs) are likely PG transporters *in vitro* (1–6), and there is published data that supports the possibility that OCTs may also play a role in PG transport (25), it remains to be clearly established whether OCTs, along with OATs, function in physiologically-relevant PG transport. Regardless, the methods we describe can be applied to many SLC22 transporters of interest and, indeed, extended to other ligand-transporter interactions. This protocol should also prove useful as a platform for studying structure-activity relationship of related substrates.

## Methods

### Materials

#### Molecular Cloning and Expression of btOCT1

Triple N-glycosylation mutant of bovine OCT1 (NP_001094568.1; N71Q, N91Q, N107Q) protein sequence was codon optimized for *Pichia pastoris* expression and the cDNA total-synthesized by GeneScript Inc. The cDNA was then subcloned into the pPICZc vector (Life Technology) between EcoRI and SalI restriction sites. A Flag tag (Sigma) and SumoStar tag (Life Sensor) were engineered sequentially N-terminal in-frame to btOCT1.

The plasmid was linearized at the PmeI restriction site and the expression host strain *P*. *pastoris* KM71H (Life Technology) transformed using the linearized plasmid according to the manufacturer’s instruction manual. The *P*. *pastoris* KM71H transformants were then screened for target expression based on signals from an anti-Flag Western blot of whole cell lysate.

BtOCT1 expressers were grown in minimal glycerol (4%) media at 28°C, supplemented with 0.4% phosphoric acid and 0.024% trace metal in Eppendorf Bio-Reactor 415 according to manufacturer’s recommendations. The pH of the media was titrated to pH 5 prior to inoculation and monitored during vegetative growth phase using 50% ammonium hydroxide. The dissolved oxygen (DO) was maintained at 10% minimally through cascaded agitation until a DO spike occurs concluding this phase. The fermentation culture was then induced at pH 5.5 with 8% methanol of final volume through a feed cascaded to DO set at 50%, 28°C overnight.

### Purification of btOCT1 protein

BtOCT1-expressing *P*. *pastoris* culture was harvested and disrupted by running the cell suspension (resuspended with lysis buffer composed of 0.1 M bis tris propane [BTP] pH 8, 0.1 M NaCl, 2 mM EDTA, 2 mM EGTA, 10% glycerol, 20 mg/l leupeptin and pepstatin A, 5 mg/l chymostatin and E-64, and 50 mM benzymidine) through a cell disruptor (Constant System) at 40,000 psi. The lysate was spun down at 12,500 g for 30 min to remove cell debris and supernatant continued onto a 38,400 g spin for 2–4 hrs to fraction the plasma membrane. The membrane fraction (MF) was resuspended into lysis buffer and frozen at -80°C for later protein preparation.

BtOCT1-containing MF suspension was solubilized with 1% βDDM and 0.25% NaCholate for 30 min at 4°C and spun down at 45,000 rpm on a 50.2 Ti rotor (Beckman Coulter) for 18 min to remove the insoluble part. Supernatant was batch bound to 5 ml of Flag resin (Sigma) for 30 min at 4°C and loaded to a gravity column. Flag resin was sequentially washed with A buffer (0.1 M BTP pH8, 0.1 M NaCl, 2 mM EDTA, and 0.05% βDDM), B buffer (0.1 M BTP pH8, 1 M NaCl, 2 mM EDTA, and 0.05% βDDM), and A buffer again before elution with 4 g/l Flag peptides in A buffer. Elution fraction was concentrated down to 0.5 ml using a 100 kD cutoff Vivaspin (GE Healthcare) concentrator before an ultracentrifugation spin at 95,000 rpm with a TLA 100.3 rotor (Beckman Coulter) for 15 min at 4°C. Supernatant from ultracentrifugation was loaded to a Superdex 200 Increase 10/300 GL gel filtration (GF) column (GE Healthcare) in GF buffer (20 mM BTP, 20 mM NaCl, and 0.05% βDDM) and the monomeric peak collected and subjected to SumoStar Protease 1 digestion (Life Sensor) for 20 min at ambient temperature. Sumostar Protease 1 digested sample was passed through Co (NiPur, Thermo Fisher) and Flag (Sigma) affinity chromatography sequentially to remove the protease and the N-terminal Flag-SumoStar tag. Flowthrough from the two tandem affinity columns contains the native btOCT1-N71/91/107Q ready for functional assays. The concentration of the native btOCT1 sample was quantified by SDS-PAGE with analytical BSA (Thermo Fisher) as standards.

### Fluorescence Polarization

BtOCT1 sample was serial 2-fold diluted with GF buffer from 3 uM for 22 times in 384-well black microtiter plate (Nunc). Five ul of 686 nM PGE2-Rhodamine conjugate (Cayman Chemical) in GF buffer was added into each well containing 25 μl btOCT1 sample. The microtiter plate was incubated at ambient temperature for 30 min before fluorescent polarization reading (Tecan Infinite 200Pro, Molecular Device FilterMax F5, or PerkinElmer Victor X5) at excitation and emission bandwidths 485 and 595 nm, respectively. FP values processed with G factor = 1 and presented as percentage ΔFP across different measurements from samples prepared independently on separate dates. Data was fitted to Hill function (y = Vmax*x^n/(k^n+x^n); where y is %FP, x is [btOCT1], Vmax = 100% FP, and n = 1) by OriginLab 7.0.

### Surface Plasmon Resonance

The btOCT1 PGE2 SPR studies were performed on a BiOptix 404pi SPR machine using either SAHC200m or SAD200m sensor chips (Xantec) made of streptavidin hydrogel. The sensor chips were pre-conditioned, and the SPR unit calibrated according to BiOpitx recommendations. Two hundred and fifty μl of the control ligand PEG11-Biotin and the test ligand PGE2-PEG11-Biotin (both Cayman Chemicals) each at 40 nM were loaded into two parallel sample flow cells on a SAHC200m chip. The surface of both control flow cells and sample flow cells were then blocked with 350 μl of 0.4% biotin and primed with GF buffer to reach a stable surface prior to btOCT1 analyte injection. A series of 40–840 nM tag-free btOCT1 samples were injected at 50 μl/min into each pair of control and sample flow cells in the 2X2 mode of the Bioptix 404pi instrument. The same btOCT1 analyte solution went through a loop of control flow cell with biotin surface only before a sample flow cell with either PEG11-Biotin control or PGE2-PEG11-Biotin immobilized sample cell surfaces, respectively. An association period of 5 min and dissociation 12 min were used. A blank buffer injection of the same volume was included before and after injection of each concentration of btOCT1 sample, for double subtraction analysis of the software (as well as thorough removal of btOCT1 from each cycle). Steady-state response deduced from Scrubber2 (BioLogic Software Pty Ltd.) analysis for both PEG11-Biotin and PGE2-PEG11-Biotin surface were plotted against analyte concentration. A Hill function fitting of the PGE2-PEG11-Biotin steady-state response using OriginLab 7.0 were performed to yield the k constant as the K_D_.

For kinetics studies of btOCT1 binding to PGE2 through the 2-over-2 strategy, 250 μl of two concentrations, 30 and 40 nM, of PGE2-PEG11-biotin were loaded at 50 μl/min onto the surface of the two separate sample flow cells of the SAD200m chip, to form two densities of ligands. Similar to the aforementioned steady state response analysis, both control flow cells and sample flow cells were then blocked by biotin and sensor chip surface primed and stabilized by GF buffer prior to analyte injection. Instead of using the same stock of btOCT1 analyte, two stepwise concentration gradients, ranging from 80 nm to 1.2 μM, were sequentially injected into each pair of control and sample flow cells in the 2X2 mode of the 404pi instrument. An association period of 5 min and dissociation 12 min were used. Also included were blank buffer injections of the same volume as the analyte solution before and after each injection of the btOCT1 sample. SPR kinetics analysis was performed using Scrubber2 with standard k_d_ and k_a_ fitting settings (one binding site) and manual bulk RI corrections to compensate responses caused by increased btOCT1 concentrations in injections. Final graphic presentation of the response data and fitted kinetic curves were presented by OriginLab 7.0.

## Results

OCT1 was first identified as an organic cation transporter and a wide range of drug and metabolite substrates have since been uncovered [11, 24]. There is good evidence supporting an important role for these transporters in normal physiology [18]. Among the highest affinity substrate interactions reported for SLC22 transporters of both the OCT and OAT families are prostaglandins, including PGE2 and PGF2a [25]. Nevertheless, the ~100–1000 fold greater affinity for prostaglandins compared to more commonly studied drug substrates raises the possibility that they may bind to different sites of the transporter and/or exert a modulatory effect on binding of other substrates. We, therefore, sought to examine direct binding to purified OCT1 transporter protein and measure association and dissociation rates using FP and SPR with two different labeled ligands.

### Purified native OCT1 is of high quality and stability

**Fig 1:**
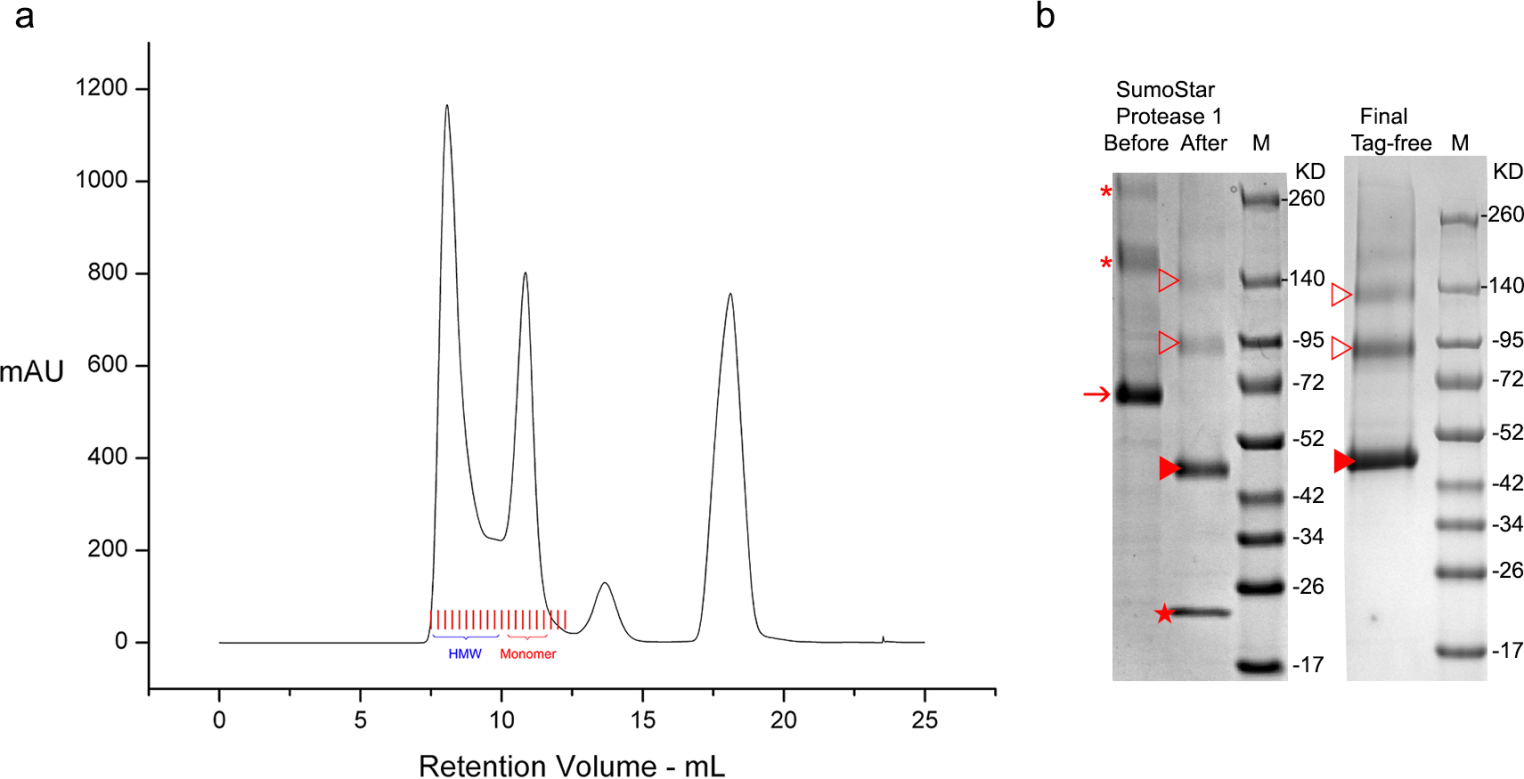
Purified native btOCT1 is greater than 99% pure. **a**. Typical size exclusion chromatography (SEC) chromatogram of affinity-purified and post-ultracentrifugation btOCT1. Red lines mark chromatography fractions (0.25 mL each) collected around btOCT1 peaks. Indicated in blue bracket are fractions having higher molecular weight (HMW) species in the Flag-affinity purified btOCT1 post ultracentrifugation pool. Monomeric fractions (indicated by red bracket) is of btOCT1 subjected to subsequent tag removal. Later elution peaks (no fraction collected) are contaminants and Flag peptides, respectively. **b**, Shows efficient expression-purification tag removal under mild conditions and the production of highly pure native btOCT1 as end product. The Flag-SumoStar tag was removed by SumoStar Protease 1 digestion after affinity and SEC chromatography at 4°C overnight in GF buffer. Closed red arrow indicates monomeric tagged btOCT1, asterisks oligomeric tagged btOCT1, closed red arrow heads monomeric tag-free btOCT1, open red arrowheads oligomeric tag-free btOCT1, red star Flag-SumoStar tag. M, molecular weight marker.

The triple N-glycosylation mutant btOCT1 is highly pure after two chromatography steps (Fig 1). The N-terminal two expression tags were removed by Sumo Star Protease 1 digestion and subsequent small-scale gravity His and Flag affinity steps. Native btOCT1 sample in the final flowthrough is >99% pure and stable at 4°C aqueous solution up to 3 days, a time window suitable for relatively long kinetics studies by SPR.

### BtOCT1 binds to PGE2-Rhodamine conjugate with submicromolar affinity

**Fig 2:**
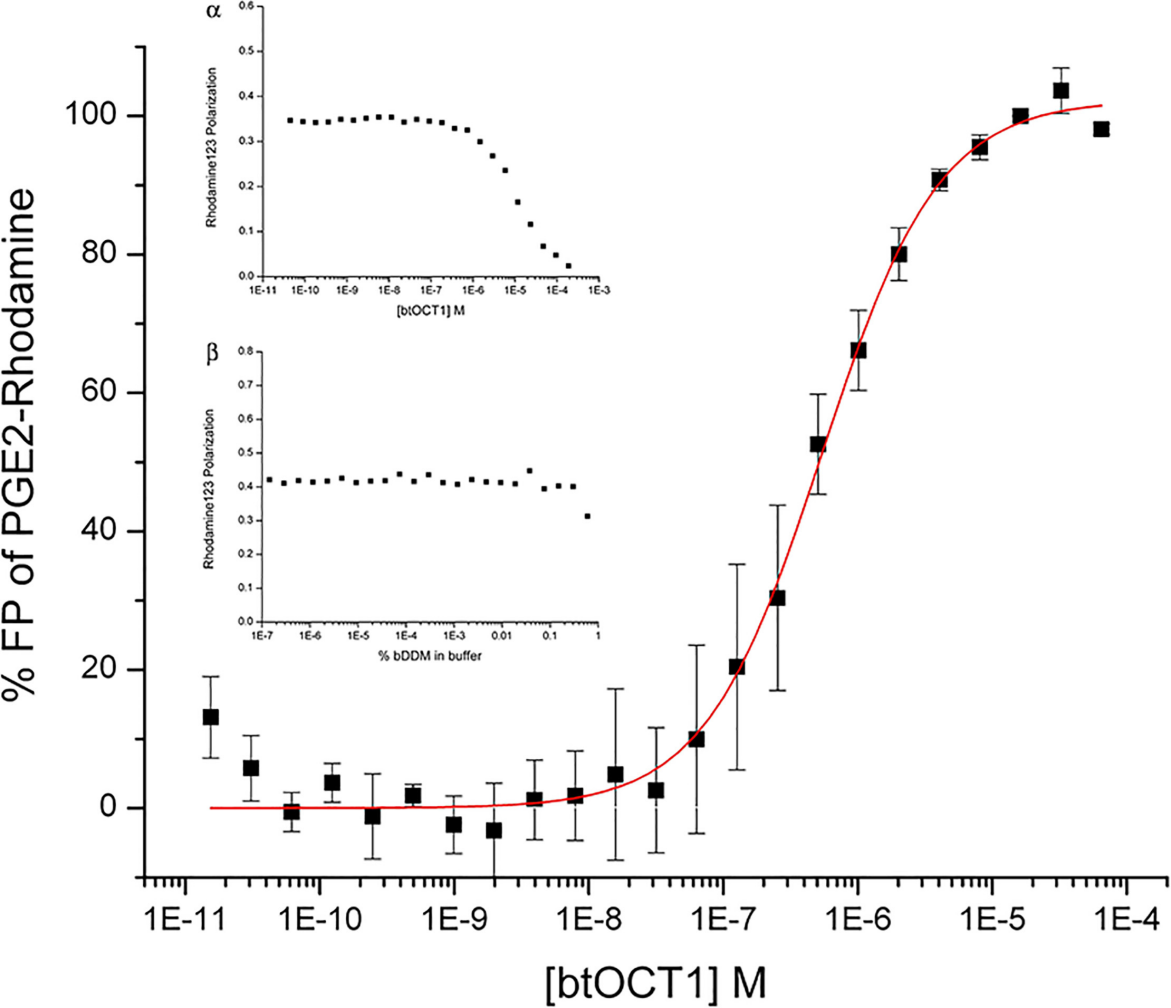
Purified btOCT1 binds to fluorescently labeled Prostaglandin E2 (PGE2-Rhodamine) with submicromolar affinity. Black closed squares are experimental data with error bar showing standard deviation between 3 independent measurements of separately prepared btOCT1 samples. Red line shows the fitted curve (Hill function) with k value = 5.404E-7 ±5.694E-8 M, representing the K_D_/affinity of btOCT1 towards the substrate PGE2. **Insert α**, shows rhodamine 123, the fluorophore of the PGE2-Rhodamine conjugate, does not show binding to similarly prepared btOCT1 protein. **Insert β** shows that the detergent used to purify btOCT1 has no consequence on the FP measurement of rhodamine 123 in the condition where the measurements were taken.

FP is a technique that has been widely utilized to investigate ligand-protein interactions, including those involving transporters [26, 27]. A fluorescently labeled PGE2 conjugate PGE2-Rhodamine was used as a probe in the OCT1-PGE2 binding study by FP. Purified btOCT1 has an affinity to PGE2-Rhodamine conjugate at 0.54 μM after curve fitting (Fig 2). The fluorosphore of the conjugate, Rhodamine 123 itself, does not bind to purified btOCT1 by the same FP analysis (Fig 2, insert α), and the buffer system in the absence of btOCT1 with detergent βDDM concentration gradient up to 0.6% does not affect the FP of the conjugate (Fig 2, insert β). The highest concentration data point of btOCT1 was estimated to contain no more than 0.4% βDDM (assuming all detergents been retained during the concentration step).

### The kinetics of label-free btOCT1 binding to immobilized PGE2 revealed slow association and dissociation rates, as well as an affinity (KD) ~100 nM

SPR has been extensively used to characterize the interactions between antibodies and antigens, as well as ligands and receptors at a high level of molecular detail. SPR kinetic curves depict the time-frame of the interaction, a useful characteristic that is often masked or imprecisely measured by other methodologies [28]. Therefore, SPR allows the discrimination of binders (eg. antibodies, agonists, antagonists), sometimes revealing dramatic differences between binders of similar affinities indicated by other methods, such as ELISA and FP.

**Fig 3:**
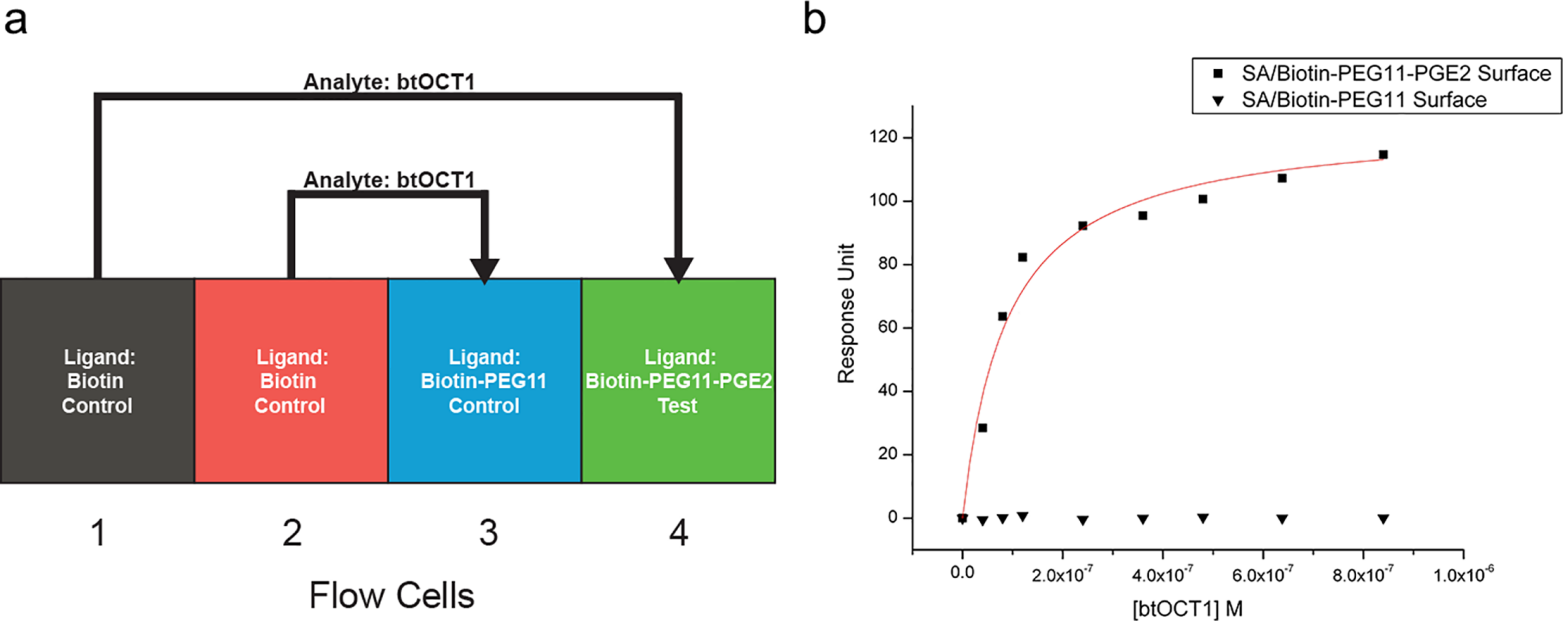
Experimental design of label-free btOCT1 binding to PGE2 by SPR. **a**, Experimental design of the SPR kinetic studies of btOCT1 binding to PGE2. Control compound PEG11-Biotin and test compound PGE2-PEG11-Biotin were immobilized on to two separate sample flow cells (3 and 4) with 3-D Streptavidin (SA) hydrogel, respectively. Blocking of the inactive sites on the flow cell surfaces using biotin and subsequent stabilization of the surface using sample buffer were performed prior to analyte injection. A 2X2 fluidics mode allows btOCT1 analyte to go through the reference flow cells (1 and 2) first before sample flow cells (3 and 4). **b**, BtOCT1 binds to immobilized PGE2 specifically. Steady-state response of btOCT1 as analyte binding to PGE2-PEG11-Biotin or PEG11-Biotin immobilized SA surfaces in parallel were plotted at closed squares and closed triangles. The k value of the fitted Hill function (red line) is 89 nM btOCT1.

For the steady-state analysis of btOCT1 PGE2 interaction, we immobilized a biotinylated PGE2 with a PEG11 spacer in between the two moieties and used the biotinylated PEG11 spacer only as control to assess the specificity of the interaction. The experimental design is shown in Fig 3A. Each sample flow cell of control and PGE2 ligand had a separate reference flow cell with only biotin immobilized on the surface, and each concentration of the same btOCT1 analyte solution as well as blank buffer injections were injected into the corresponding reference flow cell before the sample flow cell. The steady-state SPR response at the PEG11-Biotin and PGE2-PEG11-Biotin flow cell surface demonstrated that the binding of OCT1 to the flow cell surface is due to the presence of PGE2 (Fig 3B).

**Fig 4:**
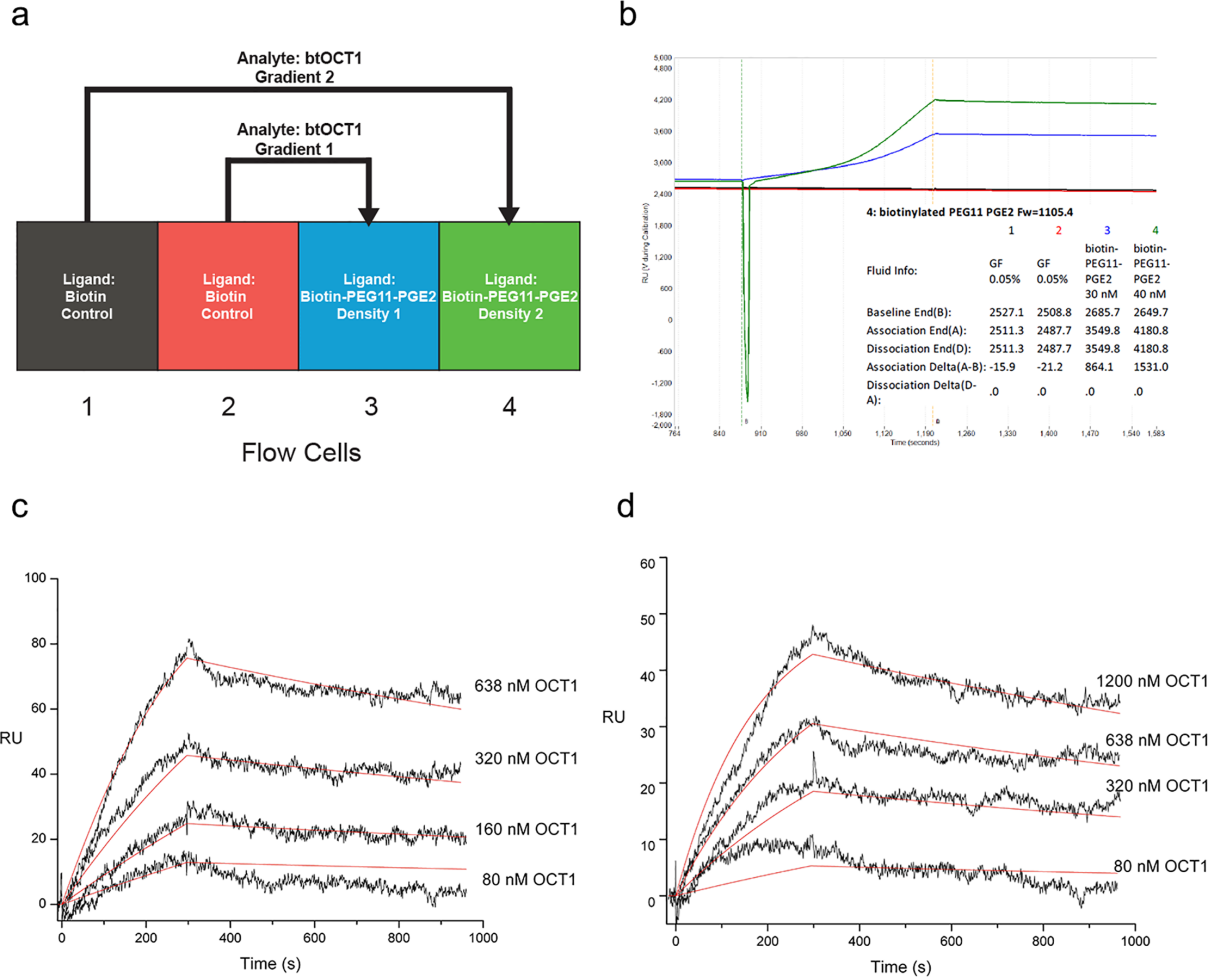
SPR kinetics of label-free btOCT1 binding to streptavidin-immobilized PGE2-PEG11-Biotin revealed a slow association and dissociation rates, as well as a binding affinity of approximately 100 nM. **a**, Experimental design of the SPR kinetic studies of btOCT1 binding to PGE2. For a 2-over-2 kinetic analysis using samples from the same preparation, both sample flow cells (3 and 4) were loaded with PGE2-PEG11-Biotin, with different concentrations to achieve different ligand density. **b.** Different ligand density on two sample flow cells (3 and 4) was achieved by using 30 nM and 40 nM, respectively, of PGE2-PEG11-Biotin during ligand loading. **c** and **d**, Two concentration series of btOCT1 as analytes binding to two surface ligand densities of PGE2-PEG11-Biotin immobilized via SA surfaces. The two parallel kinetic analyses yielded comparable K_D_ of approximately 100 nM for PGE2, with an association constant of 4837 M^-1^s^-1^ and dissociation constant of 4.69E-4 s^-1^ (Table 1). Red lines are fitted binding kinetic curves.

We then designed a 2-over-2 strategy utilizing two ligand densities on two separate sample flow cell surfaces and two series of btOCT1 analyte concentration gradients to titrate a wider range of conditions all in one experimental run (Fig 4A). The differential ligand densities on the sample cell surfaces are shown in Fig 3B. As in the steady-state analysis, each sample flow cell had a separate reference flow cell with only biotin immobilized on the surface, and each concentration of the btOCT1 analyte as well as blank buffer injections were injected into the corresponding reference flow cell before the sample flow cell, allowing double referencing for each concentration. The sensograms from the parallel sample flow cells are shown in Fig 3C and 3D. The kinetic analysis of the 2-over-2 experiment revealed a slow association (ka) and dissociation (kd) constant for the binding of btOCT1 to PGE2 (Table 1).

## Discussion

SLC22 transporters are important from a physiological, pharmacological and clinical perspective [1, 8, 9, 29]. Understanding their binding to ligands may help in the better definition of drug-drug interactions and drug-metabolite interactions as well as clarifying the effect of human mutations and polymorphisms (SNPs) on transporter function in the clinical setting. For example, some SLC22 transporter mutations are associated with metabolic disease (eg. gout, carnitine deficiency), and others are found to be associated with drug response [1]. Direct binding is generally assumed based on cell-based transport assays, but a direct analysis of the ligand/substrate with the SLC22 transporter protein needs to be demonstrated.

To our knowledge, there is no report of a structure of a mammalian SLC22 transporter and little, if any, evidence for direct binding of ligand to the transporter. One major difficulty in studying this is the relatively low affinity of most drug and metabolite substrates of multispecific drug transporters of the SLC22 family [1, 5, 6, 30].

Unusual among the likely endogenous substrates with accepted physiological importance in the organs in which SLC22 transporters are expressed are prostaglandins, including PGE2 and PGF2a [25]. In cell-based assays, affinities for prostaglandins are roughly 2–3 orders of magnitude higher than most commonly studied substrates of OCTs and OATs. Although we realize that an actual *in vivo* role for PG transport by SLC22 family members remains to be established, and even the *in vitro* transport data for certain family members such as OCT1 is limited and perhaps not conclusive, the unusually high affinity of PGE2 for OCT1 makes it a much more amenable ligand to study using techniques such as those described here. In addition, it allows one to consider whether there may be aspects of the interaction with SLC22 transporters like OCT1 that are unusual, possibly helping to explain the tight binding between prostaglandins and SLC22 transporters.

We have demonstrated the direct binding characteristics of prostaglandin analogs to pure btOCT1 protein by two different yet complementary approaches: FP and SPR. The K_D_ values obtained using both techniques are in the range of several hundred nM and are in reasonable agreement with each other, as well as previously published cell-based prostaglandin transport data [25]. The binding kinetics from SPR indicate that the relatively tight interaction between btOCT1 and PGE2 is largely contributed by a very low value dissociation rate; hence PGE2 may be different from a typical drug substrate, which are expected to exhibit fast association and dissociation rates [31]. This is further substantiated by recent studies using nucleoside reverse transcriptase inhibitors (NRTIs) with human OCTs, revealing very tight binding (K_i_ ranges of 22–500 pM) suggesting a potential role of these drugs as modulators of these transporters [32].

More biological studies are needed to conclusively establish the actual role of OCT1 in the in vitro and in vivo transport of prostaglandins as recently published cell-based transport data is not in good agreement, though this may be due to the ligand tested and the experimental conditions [33]. If OCT1 turns out not to be a transporter of PG, our binding data raises important new questions. It suggests a potentially different mode of action by PGE2, as opposed to “classical” substrates (eg. TEA), and it raises the possibility that prostaglandins could interact with OCT1 in a distinct manner from the classical substrates. Whether PGE2, as well as other structurally related PGs, binds to OCT1 at a site other than the one(s) for classical transport substrates remains to be established by detailed structural studies. It is possible that the direct high affinity interaction with the transporter protein we have demonstrated here will be helpful in efforts aimed at defining the structure of OCT1 or other SLC22 transporters in a ligand-bound state. Regardless, the tight binding of PGE2 and/or the slow dissociation rate might alter the transport function of OCT1 for physiologically-relevant and pharmacologically-important substrates.
